# Triple Threat: A Case Presentation of Pulmonary Embolism With Diabetic Ketoacidosis and Lower Respiratory Tract Infection

**DOI:** 10.7759/cureus.64364

**Published:** 2024-07-11

**Authors:** Varsha S Shinde, Yash Dixit

**Affiliations:** 1 Emergency Medicine, Dr. D. Y. Patil Medical College, Hospital and Research Centre, Dr. D. Y. Patil Vidyapeeth (Deemed to be University), Pune, IND

**Keywords:** lower respiratory tract infection (lrti), pulmonary embolism (pe), pocus (point of care ultrasound), dka: diabetic ketoacidosis, diagnostic challenge, emergency medicine

## Abstract

This case report details the diagnostic challenges and management of a middle-aged man who presented with complaints of fever and breathlessness. He was initially suspected of lower respiratory tract infection and diabetic ketoacidosis on clinical examination and treated with intravenous fluids, antibiotics, and insulin infusion. The point of care ultrasound (POCUS), as part of the primary survey, showed right atrium (RA)-right ventricle (RV) dilation and a D-shaped left ventricle, which was highly suspicious of pulmonary embolism and was later confirmed with computed tomography pulmonary angiogram (CTPA). The patient was successfully managed for pulmonary embolism, diabetic ketoacidosis, and lower respiratory tract infection.

## Introduction

Acute pulmonary embolism is a medical condition characterized by a range of symptoms, which makes it difficult to diagnose. Pulmonary embolism (PE) is a blockage in one of the lung arteries, usually caused by a blood clot that has traveled from another part of the body, often the legs. This can restrict blood flow to the lungs and is a medical emergency [1]. Diabetic ketoacidosis (DKA) is a serious condition where high blood sugar and a lack of insulin lead to the buildup of acids called ketones in the blood. This can cause dehydration, difficulty breathing, and confusion, and be life-threatening if not treated promptly. Elevated blood sugar levels, metabolic acidosis, and increased levels of ketones in the body mark diabetic ketoacidosis (DKA) [2]. It is debatable to what extent diabetes and its consequence, diabetic ketoacidosis (DKA), contribute to pulmonary thromboembolism [3]. However, it is believed to heighten the risk of severe dehydration, elevated serum viscosity, and thrombosis.

Patients with uncontrolled diabetes are more prone to lower respiratory tract infections like our patient who presented with signs and symptoms of lower respiratory tract infection (LRTI) but was eventually diagnosed with pulmonary embolism. This case presented a diagnostic challenge since it was a varied presentation of pulmonary embolism and the diagnosis of triple conditions needed a different approach. This report also mentions the utility of point-of-care ultrasound (POCUS) in the emergency medicine department and helps us in the further management of the patient. POCUS refers to a portable ultrasound device at the patient's bedside to quickly diagnose and guide treatment for various medical conditions. It also allows us to obtain real-time imaging to support immediate decision-making and management.

## Case presentation

A 45-year-old male arrived at the emergency department with progressive shortness of breath over the past three days. The symptoms began gradually and were not linked to orthopnea or paroxysmal nocturnal dyspnea. He had no history of diabetes, hypertension, asthma, tuberculosis, or previous surgeries.

On examination, the patient presented with a blood pressure of 80/50 mmHg, heart rate of 130 beats per minute, respiratory rate of 26 breaths per minute, oxygen saturation (SpO2) of 90% on room air, and cold extremities. The patient was conscious and oriented to time, place, and person. Random blood sugar was done, which was 538mg/dl (normal range: 70-110 mg/dl) and the urine dipstick test was positive for ketones (3+), which is considered large and should be negative. Arterial blood gas analysis was done, as mentioned in Table 1, giving us the diagnosis of diabetic ketoacidosis.

**Table 1:** Arterial blood gas analysis

Test	Observed value	Reference range
pH	7.28	7.35-7.45
pCO2	29 mmHg	35-45 mmHg
pO2	64 mmHg	72-104 mmHg
HCO3-	13.6 mmol/l	22-26 mmol/l
Lactate	3.3 mmol/l	Less than 2 mmol/l

**Figure 1:**
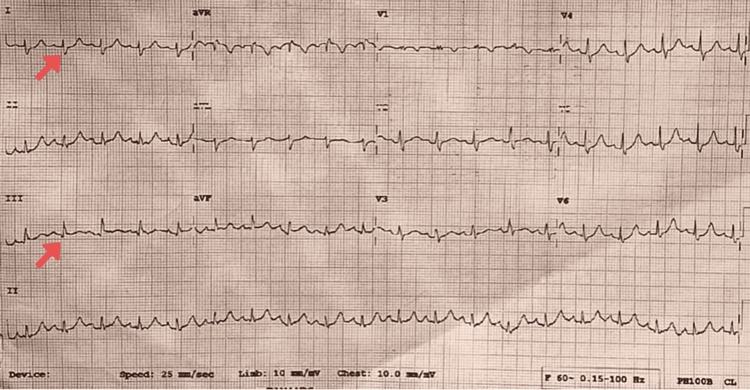
ECG shows sinus tachycardia with an S1Q3 pattern (shown with red arrows) with ST depression from V4 to V6

**Figure 2:**
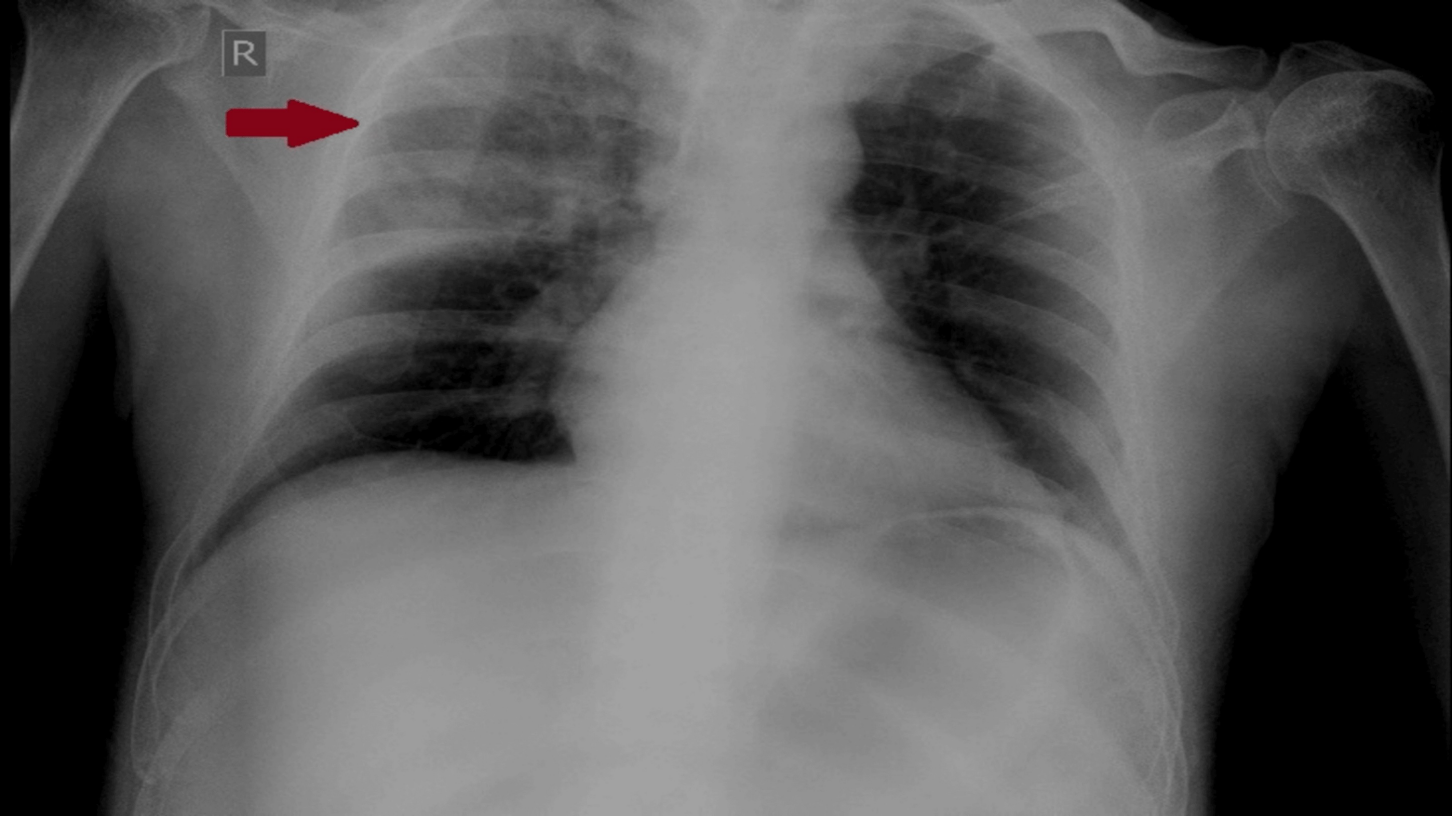
Chest X-ray showing right upper lobe opacity as depicted by the red arrow

The patient's ECG (Figure 1) indicated sinus tachycardia with an S1Q3 pattern and ST depression in leads V4 to V6, suggesting ventricular dysfunction. A chest X-ray (Figure 2) revealed opacity in the right upper lobe, suggesting a respiratory tract infection. Initial laboratory values were sent as mentioned (Table 2).

**Table 2:** Initial laboratory data of the patient INR: international normalized ratio; HbA1C: glycated hemoglobin

Test	Observed	Reference range
Hemoglobin	17.7 gm/dl	11.6-15.0 g/dl
Total leukocyte count	18800 /μL	4000-10000 /μL
Sodium	132 mmol/Lt	136 to 145 mmol/Lt
Potassium	4.13 mmol/Lt	3.50 to 5.10 mmol/Lt
Chloride	98 mmol/Lt	98 to 107 mmol/Lt
Prothrombin time	13.9 sec	10.83 to 13.17 sec
INR	1.17	0.85 - 1.15
D-dimer	>10,000 ng/ml	0 to 500 ng/ml
Trop I	1854.7 pg/ml	Up to 34.2 pg/ml
Homocysteine	58 μmol/Lt	5 to 15 μmol/Lt
Vitamin B12	<83 pg/ml	187 - 883 pg/ml
HbA1C	11.1 %	4 to 5.6 %

**Figure 3:**
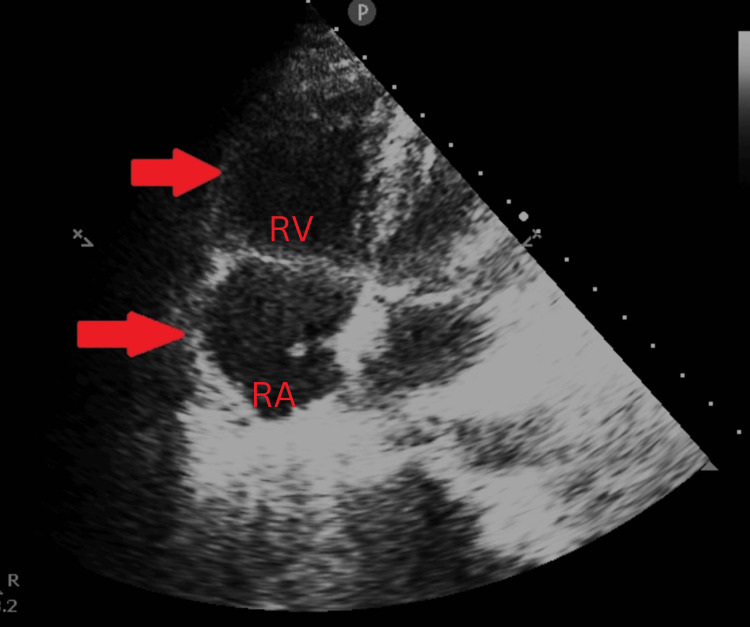
Point of care ultrasound showing the right atrium and right ventricle dilated as shown by red arrows in the apical four-chamber view

Four hours after admission, the patient’s condition deteriorated, with increased shortness of breath and the onset of chest pain. A transthoracic echocardiogram revealed a D-shaped left ventricle, enlarged right atrium and right ventricle, right ventricular dysfunction, a dilated inferior vena cava with less than 50% collapse, and a left ventricular ejection fraction of 60% (Figure 3), heightening the suspicion of pulmonary embolism.

**Figure 4:**
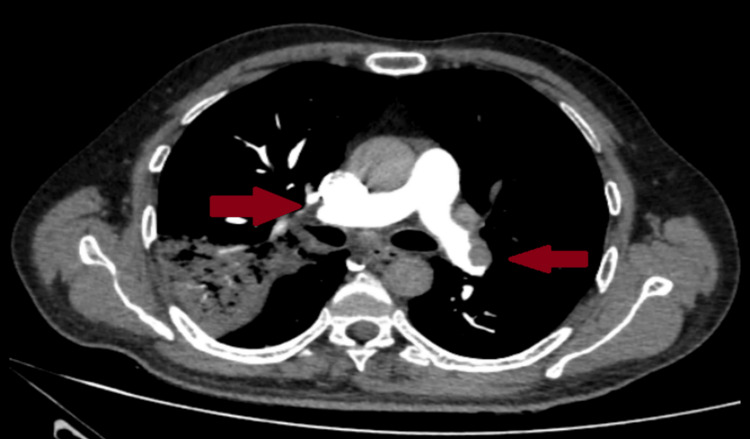
Computed tomography pulmonary angiography showing a sub-massive thrombus in major arteries as depicted by red arrows

A point-of-care ultrasound for deep venous thrombosis (DVT) was conducted and yielded no significant results. However, due to the high risk of pulmonary embolism, with point-of-care ultrasound findings and the patient's clinical findings, we proceeded with a computed tomography pulmonary angiogram. The angiogram revealed a sub-massive pulmonary thromboembolism in the major pulmonary arteries. Additionally, right upper lobe consolidation was observed, suggesting a respiratory tract infection, as depicted in Figure 4.

The patient was diagnosed with pulmonary thromboembolism with diabetic ketoacidosis with lower respiratory tract infection and treatment was started with recombinant tissue plasminogen activator, hydration, insulin, and IV antibiotics. The patient was treated successfully and discharged on oral anticoagulant and insulin.

## Discussion

Pulmonary thromboembolism can be masquerading behind diabetic ketoacidosis and lower respiratory tract infection. Our patient presented with complaints of lower respiratory tract infection but, in the end, was diagnosed and managed for diabetes ketoacidosis and pulmonary embolism.

The utility of a POCUS examination in the emergency department gives the utmost value to all emergency medicine physicians for aiding in critical diagnosis and guiding the physicians for the further approach. In our patient, POCUS gave us the picture of right heart strain, leading us toward pulmonary embolism.

In diabetic patients, the primary defense against thrombus formation is compromised due to low levels of protein C, a vitamin K-dependent plasma protein, that is crucial in regulating blood coagulation. Additionally, thrombocyte aggregation is elevated in diabetics, further increasing the risk of thrombosis [4].

Carl et al. found that individuals with DKA exhibit decreased protein C levels and increased serum fibrinogen, homocysteine, and von Willebrand factor levels [4]. Additionally, their study suggests that diabetic patients may face a higher risk of venous thromboembolism compared to nondiabetic patients [5]. High levels of homocysteine levels could also pose a risk factor for venous thromboembolism in other diabetic patients [5-6]. According to Oger et al., vitamin B12 deficiency has been identified as a risk factor for venous thromboembolism [7]. In our patient, elevated homocysteine levels were associated with this deficiency. Homocysteine, an intermediary amino acid formed during the conversion of methionine to cysteine, is metabolized through trans-sulphuration and re-methylation reactions. These metabolic processes require vitamins B6 and B12, respectively. Therefore, insufficient levels of vitamin B12 can lead to impaired homocysteine metabolism, resulting in elevated homocysteine levels, which in turn can contribute to an increased risk of venous thromboembolism [8].

Various studies have shown that point-of-care ultrasound (POCUS) is very precise in identifying cases of pulmonary embolism, with a specificity of 96.7% [9-11]. Diabetic ketoacidosis and its complications can induce a prothrombotic state, increasing the risk of pulmonary embolism for the patient. Therefore, it is essential to consider non-communicable diseases and include them in the differential diagnosis. This is particularly important for patients initially diagnosed with pneumonia and uncontrolled diabetes who experience ineffective treatment or disease progression, especially if they have no underlying health conditions.

## Conclusions

This case underscores the necessity for clinicians to maintain a high index of suspicion for pulmonary embolism even in young patients and utilize point-of-care ultrasound in the emergency department for timely intervention in managing such cases. Point-of-care ultrasound is an increasingly utilized diagnostic tool that offers several advantages, particularly in situations where time and cost are critical factors, ultimately improving patient outcomes and reducing the morbidity and mortality associated with this potentially life-threatening condition.

This case highlights the importance of considering pulmonary thromboembolism in patients with diabetic ketoacidosis, especially when presenting with respiratory symptoms. This patient was successfully managed in the emergency department and was later discharged on anticoagulants and insulin.
